# Pharmacological profiling of the hemodynamic effects of cannabinoid ligands: a combined in vitro and in vivo approach

**DOI:** 10.1002/prp2.143

**Published:** 2015-05-08

**Authors:** Sarah K Walsh, Claire Y Hepburn, Oliver Keown, Annika Åstrand, Anna Lindblom, Erik Ryberg, Stephan Hjorth, Stephan J Leslie, Peter J Greasley, Cherry L Wainwright

**Affiliations:** 1 Institute for Health & Wellbeing Research, Robert Gordon UniversityRiverside East, Aberdeen, AB10 7GJ, United Kingdom; 2 Cardiovascular & Metabolic Disease IMED, AstraZeneca R&DMölndal, Sweden; 3 Cardiac Unit, Raigmore HospitalOld Perth Road, Inverness, IV2 3UJ, United Kingdom

**Keywords:** Cannabinoids, CB_1_ receptor, G protein coupled receptor 55, hemodynamics

## Abstract

The receptors mediating the hemodynamic responses to cannabinoids are not clearly defined due to the multifarious pharmacology of many commonly used cannabinoid ligands. While both CB_1_ and TRPV1 receptors are implicated, G protein-coupled receptor 55 (GPR55) may also mediate some of the hemodynamic effects of several atypical cannabinoid ligands. The present studies attempted to unravel the pharmacology underlying the in vivo hemodynamic responses to ACEA (CB_1_ agonist), O-1602 (GPR55 agonist), AM251 (CB_1_ antagonist), and cannabidiol (CBD; GPR55 antagonist). Agonist and antagonist profiles of each ligand were determined by ligand-induced GTP*γ*S binding in membrane preparations expressing rat and mouse CB_1_ and GPR55 receptors. Blood pressure responses to ACEA and O-1602 were recorded in anesthetized and conscious mice (wild type, CB_1_^−/−^ and GPR55^−/−^) and rats in the absence and presence of AM251 and CBD. ACEA demonstrated GTP*γ*S activation at both receptors, while O-1602 only activated GPR55. AM251 exhibited antagonist activity at CB_1_ and agonist activity at GPR55, while CBD demonstrated selective antagonist activity at GPR55. The depressor response to ACEA was blocked by AM251 and attenuated by CBD, while O-1602 did not induce a depressor response. AM251 caused a depressor response that was absent in GPR55^−/−^ mice but enhanced by CBD, while CBD caused a small vasodepressor response that persisted in GPR55^−/−^ mice. Our findings show that assessment of the pharmacological profile of receptor activation by cannabinoid ligands in in vitro studies alongside in vivo functional studies is essential to understand the role of cannabinoids in hemodynamic control.

## Introduction

The in vivo cardiovascular effects of cannabinoids, the most studied of which is the endocannabinoid anandamide (AEA), are complex and vary substantially depending upon the experimental design and whether or not the studies are performed in anesthetized or conscious animals (reviewed in Malinowska et al. 2012). Moreover, responses are mediated through a mixture of direct vasodilator effects on blood vessels, a reduced cardiac contractility, and modulation of autonomic control of both the heart and the vasculature. In anesthetized rats, intravenous (i.v.) administration of AEA elicits a three phase blood pressure (BP) response involving the transient receptor potential vanilloid-1 (TRPV1) receptors located on sensory vagal nerves in the heart (phase 1 transient depressor and phase 2 transient pressor responses) and CB_1_ receptors present in the vasculature and myocardium (phase 3 prolonged fall in BP; Varga et al. 1996; Lake et al. 1997; Malinowska et al. 2001). In contrast, in conscious normotensive rats, AEA causes a pressor response (Gardiner et al. 2002), whilst in conscious spontaneously hypertensive rats (SHR) the initial two phases of the classical three phase response are followed by prolonged vasodilation (Ho and Gardiner 2009).

However, there are studies that suggest other receptors are involved in mediating these responses, and this also holds true for other cannabinoid ligands. For example, evidence exists for the presence of an endothelial SR141716-sensitive non-CB_1_, non-CB_2_, non-vanilloid receptor that is unresponsive to certain established CB_1_/CB_2_ receptor agonists such as WIN-55,212-2, but can be activated by both AEA and its stable analog methanandamide, as well as lipid conjugates such as virodhamine and *N*-arachadonoylglycine (NAGly) (Ho and Hiley 2004; Parmar and Ho 2010). A further receptor that has been proposed to be activated by AEA is the G-protein-coupled receptor 55 (GPR55), which belongs to a group of rhodopsin-like seven transmembrane/G-protein-coupled receptors and was originally isolated in human caudate nucleus (Sawzdargo et al. 1999). Some of our group (Ryberg et al. 2007) were the first to describe the ligand pharmacology of this receptor and demonstrated that AEA, virodhamine, the abnormal cannabidiol (abn-CBD; a synthetic regioisomer of CBD), and its synthetic analog, O-1602 all bind to and activate the receptor in vitro*,* findings that have been corroborated by further studies (e.g., Waldeck-Weiermair et al. 2008), thus adding support to the proposal that GPR55 is a putative third cannabinoid receptor (Baker et al. 2006; Pertwee 2007).

While the receptors involved in mediating BP responses to endocannabinoids remain to be determined, the picture is further complicated by the complex and multifarious pharmacology of the compounds used as experimental tools to address the question. For example, AM251 was originally described as a selective CB_1_ antagonist (reviewed in Howlett et al. 2002), but it is now recognized that it also acts as a GPR55 agonist (Ryberg et al. 2007; Pertwee et al. 2010). In addition, the phytocannabinoid CBD has routinely been used as a GPR55 antagonist (Ryberg et al. 2007), yet it also demonstrates activity at various other receptors (Pertwee 2008; Pertwee et al. 2010). Similarly O-1602, which was initially regarded and utilized as a GPR55 agonist since its actions can be blocked by CBD (Jarai et al. 1999)is now thought to exert equipotent action at another orphan cannabinoid candidate receptor (GPR18; McHugh et al. 2010).

Most previous studies have focused on either the in vitro assessment of the ability of ligands to bind to CB_1_ or GPR55 receptors and activate downstream signaling processes (e.g., Ryberg et al. 2007; Pertwee et al. 2010; Henstridge et al. 2011) or on their vascular functional responses (e.g., Wagner et al. 1999; Ho and Hiley 2003, 2004), but rarely both. Therefore, this study was performed to first determine the receptor profiles of CBD, AM251, O-1602, and the CB_1_ agonist arachidonyl-2′-chloroethylamide (ACEA) using a GTP*γ*S-binding assay in membrane preparations exclusively expressing recombinant CB_1_ or GPR55 receptors. Once receptor profiles were identified, we then studied the hemodynamic responses to each of these compounds in both anesthetized and conscious rats and mice, using wild type, CB_1_, and GPR55 knockout mice, to determine whether the in vivo hemodynamic profiles correlated with the activation profiles determined in vitro.

## Materials and Methods

### Assessment of ligand-induced GTP*γ*S binding in membrane preparations expressing rat and mouse CB_1_ and GPR55 receptors

HEK293s cells were transiently transfected with cDNA encoding rat or mouse CB_1_ or GPR55 and membranes were prepared as previously described (Ryberg et al. 2007). In short, agonist activities in [^35^S]-GTP*γ*S binding assays were determined by the addition of ligand at 30°C for 45 min in membrane buffer [50 mmol/L NaCl, 5 mmol/L MgCl_2_, 0.5 mmol/L EDTA, 25 mmol/L HEPES (all Sigma, Gillingham, Dorset, UK); pH 7.4] containing 0.05 *μ*g *μ*L^−1^ of membrane protein with 0.01% BSA, 25 *μ*mol/L guanosine 5′-diphosphate (GDP), 100 *μ*mol/L dithiothreitol (DTT), and 0.53 nmol/L [^35^S]GTP*γ*S (PerkinElmer) in a final volume of 200 *μ*L. Antagonist assays were performed similarly, with the addition of an EC_80_ concentration of CP55940 as a nonselective CB_1_/CB_2_ agonist in conjunction with the ligand to be tested. Nonspecific binding was determined in the presence of 20 *μ*mol/L unlabeled GTP*γ*S. The reaction was terminated by addition of ice-cold wash buffer (50 mmol/L Tris-HCl, 5 mmol/L MgCl_2_, 50 mmol/L NaCl; pH 7.4) followed by rapid filtration under vacuum through 96-well B-glass fiber filter plates (PerkinElmer, Waltham, Massachusetts, USA) using a Biomek FX (Beckman Coulter, Bromma, Sweden). The filter plates were dried (30 min at 50°C) before scintillation liquid (PerkinElmer) was added onto the filters and the bound radioactivity was determined using a scintillation counter (Wallac). Data were fitted with a four-parameter logistic fit using the equation *y* = *A* + (*B* − *A*)/1 + ((*C*/*x*)^*D*), where *A* is no activation, *B* is full activation, *C* is the EC_50_, and *D* is the Hill slope. All data were based on at least three independent experiments.

### In vivo studies

#### Animals

All procedures were performed under either a Project Licence issued under the UK Animals (Scientific Procedures) Act 1986 (procedures under terminal anesthesia) or under the approval of the local Ethical committee in Gothenburg (conscious studies). The studies were designed to comply with the ARRIVE Guidelines for reporting in vivo studies (Kilkenny et al. 2010). Normotensive Sprague–Dawley (SD) rats and SHR were purchased from Charles River (Tranent, Scotland, UK). CB_1_ knockout (CB_1_^−/−^) mice and their corresponding wild-type (WT; C57Bl/6J) control were bred at AstraZeneca (AZ) from founder pairs obtained from Dr. Andreas Zimmer, Germany (Zimmer et al. 1999). GPR55 knockout (GPR55^−/−^) mice were bred at both AZ and the University of Aberdeen from heterozygous GPR55 knockout mice, which were intermated to generate F1 mice homozygous for the GPR55 mutation (GPR55^−/−^) and WT (C57Bl/6J) littermate controls. Both males and females were used and genotyped as previously described (Whyte et al., 2009). Neither genetically modified strain demonstrated any obvious phenotypic difference from WT animals. All animals were group housed in cages at a temperature of 21 ± 2°C and 55 ± 10% humidity with a 12 h light/dark cycle and allowed free access to food and tap water.

#### Hemodynamic studies in anesthetized rats and mice

All experiments performed under terminal anesthesia were carried out following approval by Robert Gordon University Animal Ethics Panel. Anesthesia was induced in male SD rats (200–300 g) by sodium pentobarbital [60 mg kg^−1^ intraperitoneal (i.p.) injection]. Male/female WT and GPR55^−/−^ mice (25–35 g) were anesthetized with a mixture of ketamine (120 mg kg^−1^) and xylazine (16 mg kg^−1^) via i.p. injection. For both species the trachea was cannulated to allow artificial respiration with room air when required (54 strokes min^−1^ and tidal volume 1.5 mL 100 g^−1^ for rats; 120 strokes min^−1^ and tidal volume 100 *μ*L 10 g^−1^ for mice) and the left carotid artery and right jugular vein were cannulated for the measurement of arterial BP (MLT844 Physiological Pressure Transducer; AD Instruments, Oxford, UK) and drug administration, respectively. Core temperature was monitored continuously and maintained at 37–38°C with the aid of a Vetcare heated pad (Harvard Apparatus Ltd., Boston, Massachusetts, USA). Anesthesia was monitored by foot pinch reflex and maintained throughout by administration of additional doses of anesthetic (rats: 3–4 mg kg^−1^ of sodium pentobarbital salt i.v.; mice: 50 *μ*L 25 g^−1^of ketamine/xylazine i.p.) every 40 min, or as required. Both mean arterial blood pressure (MABP) and a standard limb lead I electrocardiogram (ECG) were monitored continuously throughout the experimental period using a Power Lab data acquisition system via a Bridge Amplifier and Animal Bio Amplifier, respectively, and data subsequently analyzed using Chart Software (all AD Instruments). Heart rate (HR) was calculated from the ECG and recorded throughout the entire experimental period. After a stabilization period of approximately 15 min dose–response experiments were carried out as described below.

#### Blood pressure measurements in conscious rats and mice

All studies in conscious animals were performed in SHR. Following anesthesia with isoflurane (4% in air followed by 2% during surgery) with supplemental Romefen® (10 mg kg^−1^ s.c.; Merial SAS, Lyon, France) for analgesia, a polyethylene catheter (PE10) was inserted into the abdominal aorta, proximal to the kidneys, for BP recording, and a silicon catheter (SEDAT, Irigny, France) was inserted into the jugular vein for drug administration. Both catheters were tunneled subcutaneously to the neck region and anchored to an inert button. Immediately after completion of surgery the rats were connected to a tethered swivel system with a continuous intra-arterial infusion (700 *μ*L h^−1^) of 0.9% NaCl containing melagatran (10 mg L^−1^) in order to keep the arterial line open. The anesthetic and surgical procedures for the CB_1_^−/−^, GPR55^−/−^, and WT mice used for the studies in conscious animals were identical to those in the rat, with the exception that the arterial catheter was placed in the descending aorta via the carotid artery. Following the completion of surgery, the animals were allowed to recover for 24 h with free access to food and water. During the experiments, both BP and HR were recorded continuously in conscious animals and data fed into in-house software (v.4.0; PharmLab), reporting average values of MABP, systolic, diastolic, and pulse pressures, HR and body temperature every 15 sec.

### Experimental protocols

#### Assessment of the hemodynamic responses to ACEA in the absence and presence of AM251 and CBD

**Figure 1:**
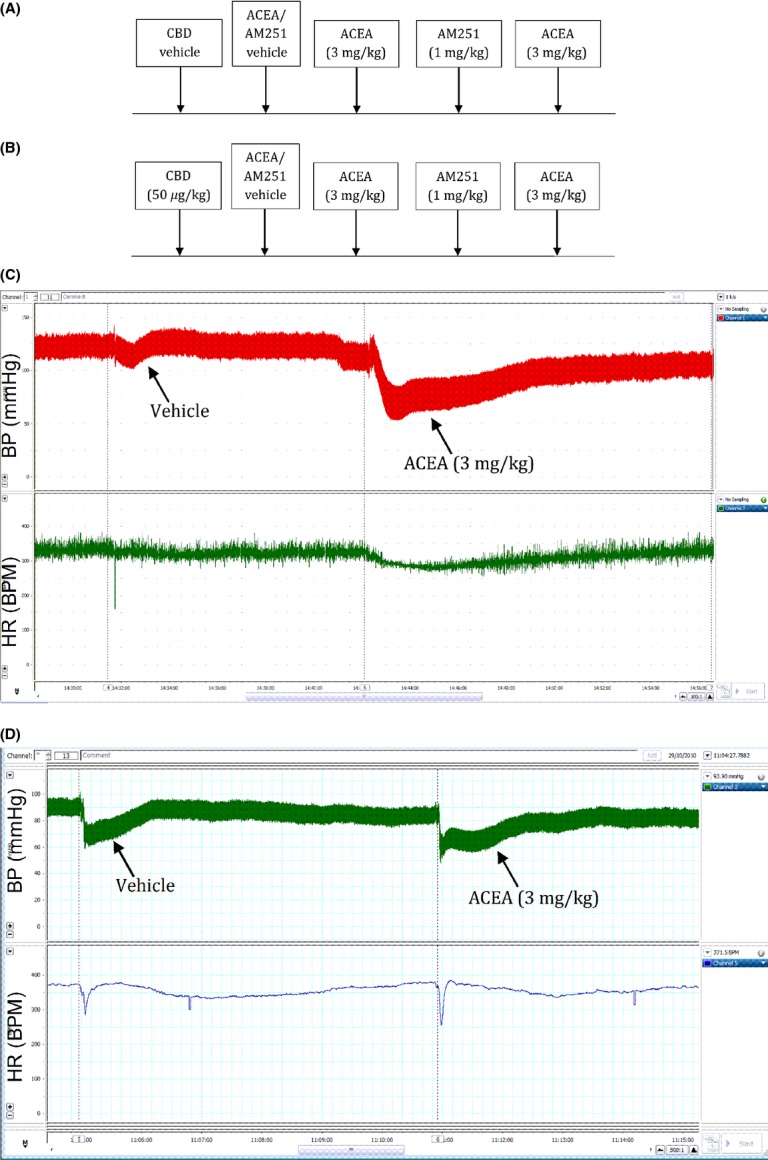
Experimental protocol for the assessment of the effects of AM251 on the depressor responses to ACEA in anesthetized rats and mice. Experiments were performed in the absence (A) or presence (B) of CBD (50 *μ*g kg^−1^) in separate groups of animals. Examples of original traces showing the blood pressure (top trace) and heart rate (bottom trace) responses to ACEA and its vehicle in rats (C) and mice (D).

As a preliminary assessment of the effects of ACEA on arterial BP, a single bolus intravenous dose (3 mg kg^−1^; selected from the literature as a dose known to produce a depressor response) was given to anesthetized rats. This was then repeated in the presence of AM251 (1 and 3 mg kg^−1^), CBD (50 *μ*g kg^−1^) or a combination of the two to establish their ability to influence the response to ACEA (See Fig.1 for the Experimental protocols). Since the data from the GTP*γ*S-binding assay (Table1) demonstrated ACEA to exhibit activity at GPR55 within the nanomolar range (and only one order of magnitude higher than at CB_1_ receptors) we also explored the role of GPR55 in the depressor response to ACEA in anesthetized WT and GPR55^−/−^ mice using the same experimental protocol as that described for the anesthetized rats (Fig.1). To account for any vehicle effects, all responses to ACEA were assessed either in the presence of these antagonists or their vehicles (CBD dissolved in ethanol; AM251 dissolved in a mixture of DMSO and Tween 80; both were diluted with saline prior to drug administration; Fig.1). The time interval between drug administrations was 10–15 min to allow BP values to return to predrug values.

**Table 1:** Ligand-dependent [^35^S]-GTP*γ*S binding assays performed in membranes prepared from HEK293s cell transiently transfected with either rat or mouse CB_1_ or GPR55 cDNA

	CB_1_ EC_50_ (nmol/L)		GPR55 EC_50_ (nmol/L)	
Compound	Rat	Mouse	Rat	Mouse
ACEA	7 ± 1	3 ± 0.5	78 ± 4	62 ± 3
O-1602	>30,000	>30,000	20 ± 3	12 ± 4
CBD	>30,000	>30,000	624 ± 13*	780 ± 8*
AM251	26 ± 8*	13 ± 2*	74 ± 3	68 ± 3
AM281	13 ± 4*	8 ± 1*	>30,000	>30,000
AEA	34 ± 2	25 ± 8	24 ± 3	38 ± 3

EC_50_ values are expressed as mean ± SEM of at least three independent experiments.

“^*^” indicates that the ligand behaved as an antagonist.

#### Assessment of the hemodynamic responses to O-1602

Using an identical protocol to that used for ACEA, the effect of O-1602 (5–100 ng kg^−1^ administered as incremental i.v. doses) was determined on HR and BP in anesthetized rats (in the absence and presence of AM251 and/or CBD) and in anesthetized WT and GPR55^−/−^ mice. Since we were unable to observe any hemodynamic responses to O-1602 compared to vehicle (methyl acetate) in anesthetized animals, these experiments were repeated in conscious mice to abrogate any effects of anesthesia on the hemodynamic responses, using a higher dose range of O-1602 (5, 10 and 15 mg kg^−1^ i.v.). To determine any role for CB_1_and GPR55, these experiments were performed in WT, GPR55^−/−^ and CB_1_^−/−^mice; for the studies in WT and CB_1_^−/−^ mice, responses to O-1602 were determined in the absence (vehicle) and presence of CBD (5 mg kg^−1^), whereas in the GPR55^−/−^ mice responses were determined in the absence (vehicle) and presence of the CB_1_ antagonist AM281 (10 mg kg^−1^). Finally, since the cardiovascular effects of cannabinoids can be exaggerated in hypertension (Ho and Gardiner 2009), we investigated whether a depressor response to O-1602 could be unmasked in the setting of elevated MABP and intact autonomic vascular control by using SHR’s pretreated with metoprolol (10 mg kg^−1^ i.v.), to prevent baroreceptor reflex correction of changes in BP.

#### Assessment of the hemodynamic responses to AM251 and CBD

Although the study was originally designed to determine the ability of CBD and AM251 to block the depressor responses to ACEA and O-1602, both CBD and AM251 were found to produce depressor responses themselves which were worthy of study, particularly since AM251 has been reported to possess pharmacological actions at both CB_1_ (as an antagonist) and GPR55 (as an agonist) receptors (Ryberg et al. 2007; reviewed in Stanley et al. 2012).For CBD, anesthetized rats and mice (both WT and GPR55^−/−^) were administered a single bolus dose of CBD (50 *μ*g kg^−1^). For AM251 similar experiments were performed, but in the absence and presence of CBD to determine whether CBD mimicked the effects of GPR55 deletion.

#### Measurement of hemodynamic responses and statistical analyses

In order to take account of any vehicle responses and variations in the time course of the responses to the various cannabinoid ligands, a standard approach was adopted to measuring the BP responses in all experimental models. MABP measurements were taken every 15 sec from 1 min prior to drug/vehicle until 10 min postdrug administration. Changes in MABP at each sampling point were calculated as a % change in MABP and time plots used to calculate area above the curve (AAC) for depressor responses or area under the curve (AUC) for pressor responses. Data were expressed as mean ± SEM. and MABP responses compared directly by either a paired *t*-test (responses within animals) or by two-way analysis of variance (ANOVA) followed by Bonferroni post hoc test (for comparing multiple drug interventions across several groups). All drug responses were compared directly with the vehicle responses to determine the exact magnitude of the response to the ligand.

## Results

### [^35^S]-GTPγS binding assays

The EC_50_ data for all of the ligands used in the [^35^S]-GTP*γ*S binding assays are shown in Table1. ACEA demonstrated GTP*γ*S binding at both receptors, with an approximately 10-fold higher affinity for CB_1_ in both mouse and rat tissue as compared to GPR55.O-1602-activated GPR55 with a potency approximately 5 times that of ACEA. AM251 exhibited significant activity at both CB_1_ (as an antagonist) and GPR55 (as an agonist), with only a three to five-fold higher potency for CB_1_. In contrast, AM281 demonstrated antagonist activity selectively for CB_1_ and CBD demonstrated selective antagonism at GPR55. For comparison, AEA was also included in the binding assay and exhibited similar activity at both CB_1_ and GPR55 in the low nanomolar range consistent with previous findings for the human receptor (Ryberg et al. 2007). In conclusion, there were no significant species differences in EC_50_ values for either receptor for any of the ligands tested.

### Hemodynamic responses to ACEA in normotensive anesthetized rats and mice

**Figure 2:**
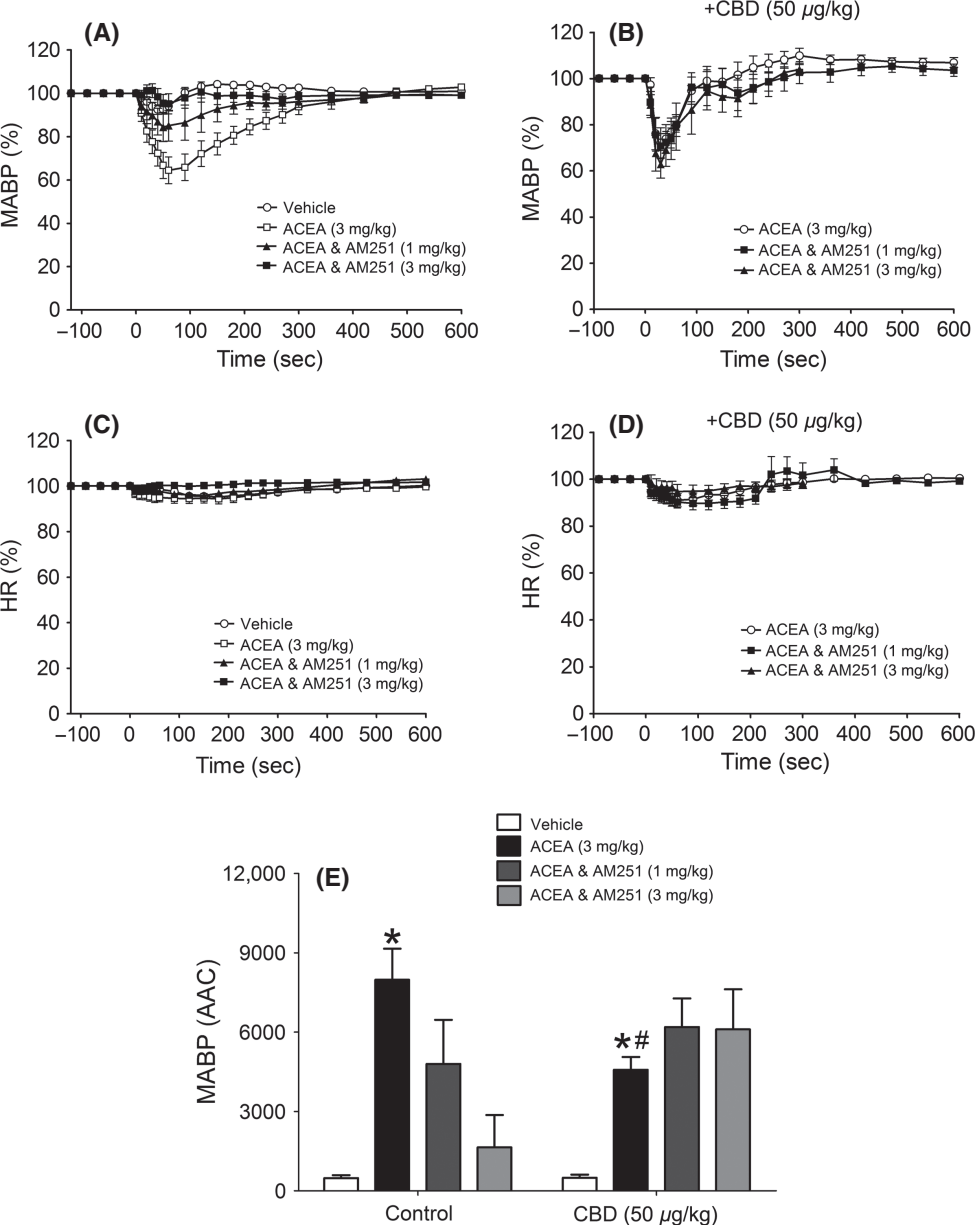
Hemodynamic responses to ACEA and its vehicle in normotensive anesthetized rats, showing the time course of the depressor responses (expressed as a percentage fall in mean arterial blood pressure from baseline) in the absence (A) and presence (B) of CBD (50 *μ*g kg^−1^) and percent changes in heart rate (C and D). Baseline MABP’s and HR’s for each group were control (129 ± 4 mmHg and 379 ± 8 bpm; *n* = 8) and CBD (135 ± 4 mmHg and 390 ± 4 bpm; *n* = 8), respectively. Panel (E) summarizes the mean areas above the curve for the blood pressure response (AAC in arbitrary units). All values shown are mean ± SEM; *P <* *0.01 versus vehicle (within group); ^#^*P *<* *0.05 versus ACEA (control group).

**Figure 3:**
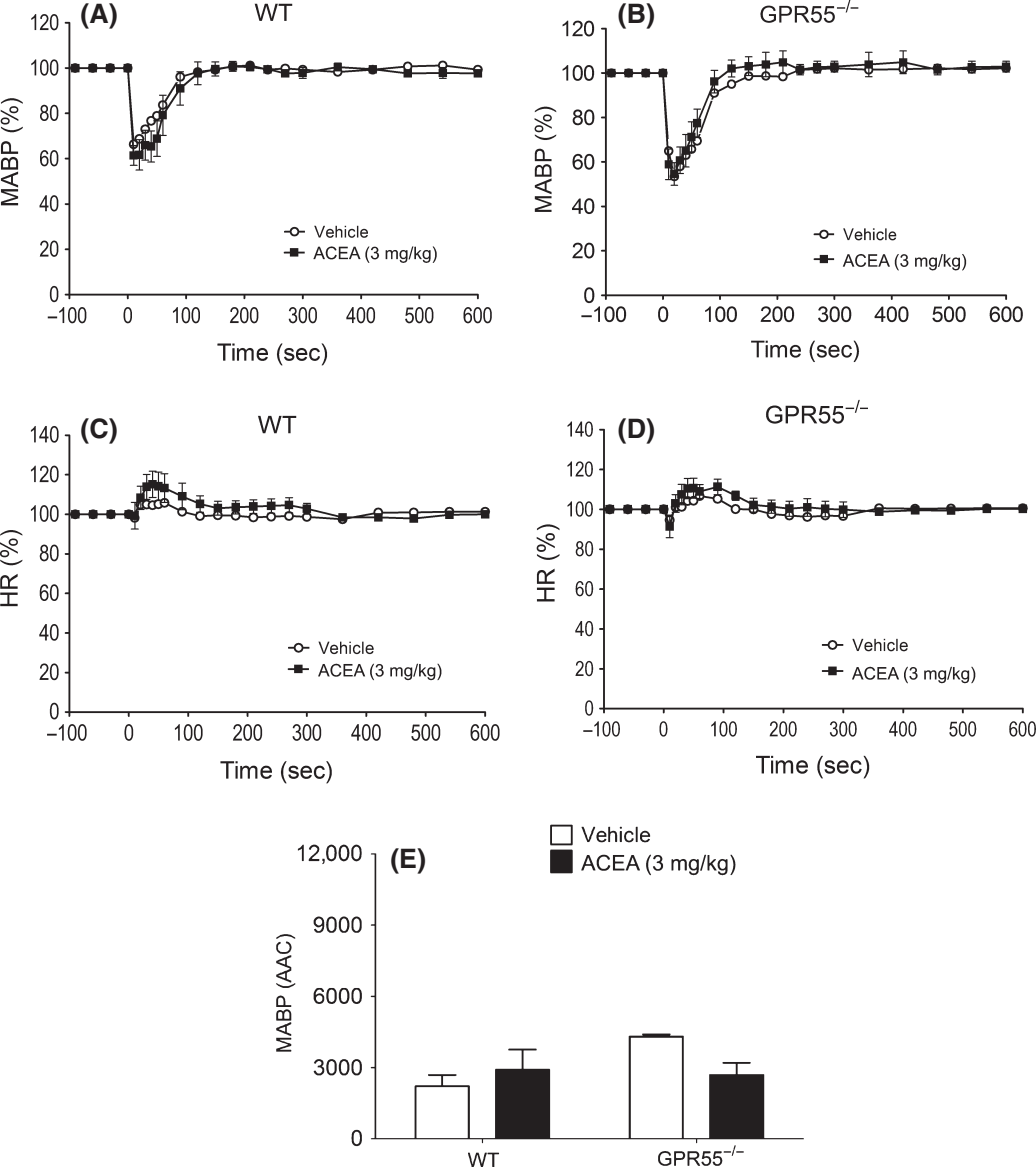
The time course of the depressor responses (expressed as a percentage fall in mean arterial blood pressure from baseline) to ACEA and its vehicle in WT (A) and GPR55^−/−^ (B) mice and percent changes in heart rate (C and D). Baseline MABP’s and HR’s for each group were WT (89 ± 3 mmHg and 329 ± 6 bpm; *n* = 8) and GPR55^−/−^ (89 ± 2 mmHg and 339 ± 5 bpm; *n* = 8), respectively. Panel (E) summarizes the mean areas above the curve for the blood pressure response (AAC in arbitrary units). All values shown are mean ± SEM.

A single bolus dose of ACEA (3 mg kg^−1^) produced a reproducible and pronounced depressor response in anesthetized rats (*P *<* *0.001; Figs.2A and E), but had no effect on HR (Fig.2C). The magnitude and duration of the response to ACEA was attenuated by AM251 (1 and 3 mg kg^−1^) in a dose-dependent manner (*P *<* *0.01; Figs.2A and E). In the presence of CBD alone (50 *μ*g kg^−1^) the duration of the response to ACEA was blunted and consequently the AAC value was reduced by approximately 50% (*P *<* *0.05; Figs.2B and E). Combined administration of CBD followed by AM251 5 min later, did not produce an additive blockade of the ACEA response; indeed the blockade seen with the higher dose of AM251 was no longer evident (Fig.2E). In contrast, ACEA did not produce a depressor response in anesthetized mice that could be distinguished from the response to the vehicle in either WT (Fig.3A) or GPR55^−/−^ (Fig.3B) mice, nor was there any measurable effect on HR (Figs.3C and D).

### Hemodynamic responses to O-1602

**Figure 4:**
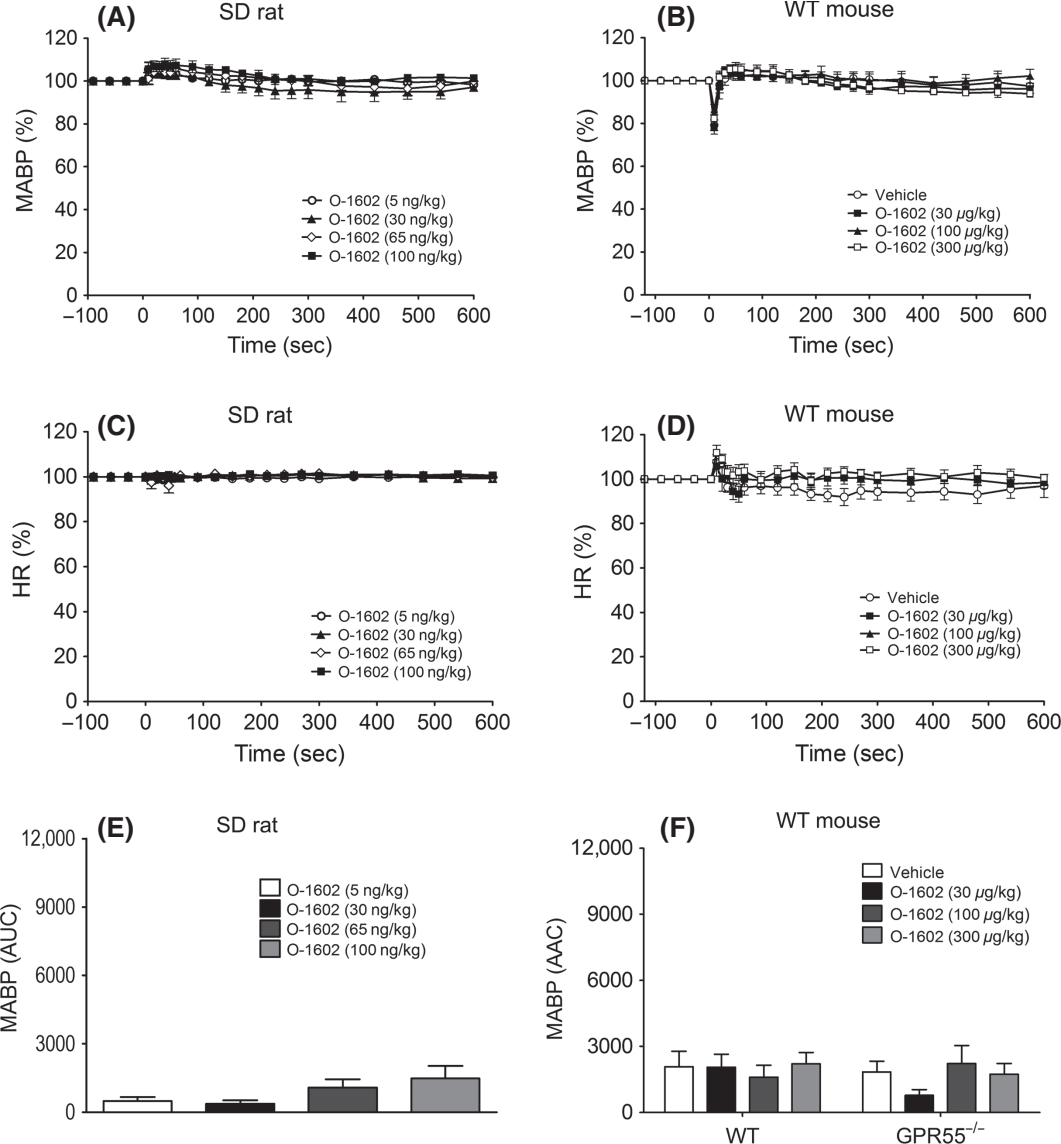
Blood pressure and heart rate responses to O-1602 (5–100 ng kg^−1^) in normotensive anesthetized rats (A and C) and WT mice (B and D). Baseline MABP’s and HR’s for each group were SD rats (133 ± 2 mmHg and 433 ± 9 bpm; *n* = 8) andWT mice (90 ± 3 mmHg and 334 ± 6 bpm; *n* = 8), respectively. Panels (E and F) show the area above the curve (arbitrary units) for the blood pressure response in rats and mice respectively. Values shown are mean ± SEM.

**Figure 5:**
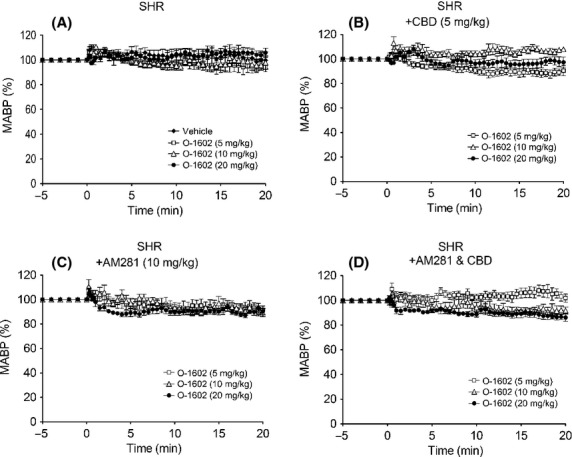
Blood pressure and heart rate responses to O-1602 (5–20 mg kg^−1^) in hypertensive SHR rats in the presence of vehicle (A); CBD (5 mg kg^−1^; B); AM281 (10 mg kg^−1^; C); or a combination of CBD and AM281 (D). Baseline MABP’s and HR’s for each group were SHR (146 ± 5 mmHg and 305 ± 10 bpm; *n* = 6); SHR & CBD (141 ± 5 mmHg and 296 ± 8 bpm; *n* = 6); SHR &AM281 (153 ± 6 mmHg and 296 ± 9 bpm; *n* = 7); and SHR & CBD& AM281 (151 ± 5 mmHg and 297 ± 12 bpm; *n* = 7), respectively. Values shown are mean ± SEM.

O-1602 (5–100 ng kg^−1^) did not produce any changes in MABP or HR in anesthetized rats (Figs. 4A, C, and E) or WT mice (Figs.4B, D, and F) over and above those seen with the vehicle. In conscious hypertensive rats, O-1602 administered in three ascending doses (5, 10, and 20 mg kg^−1^) spaced 20 min apart did not induce any changes in MABP (Fig.5A) or HR (Table2), even though O-1602 was given at much higher doses than those given to anesthetized rats. However, CBD pretreatment revealed a vasodepressor response to the lowest dose of O-1602 (Fig.5B, Table2) while co-administration of 10 mg kg^−1^ AM281 (a more selective CB_1_ antagonist; Ryberg et al. 2007) revealed a marked vasodepressor response, which was present for all doses and was sustained in the presence of 5 mg kg^−1^ CBD (Figs.5C and D). None of the interventions induced any changes in HR (Table2).

**Table 2:** Hemodynamic responses to O-1602 in conscious hypertensive rats, pretreated with metoprolol (10 mg kg^−1^ i.v.) to prevent baroreceptor reflex correction of blood pressure, in the absence or presence of AM281 (10 mg kg^−1^), CBD (5 mg kg^−1^) or a combination of the two

MABP [depressor response - area above the curve (arbitrary units)]	Control	+CBD	+AM281	+CBD and AM281
Vehicle	22 ± 19	–	–	–
O-1602 (5 mg kg^−1^)	59 ± 21	189 ± 32*	196 ± 44*	41 ± 17
O-1602 (10 mg kg^−1^)	56 ± 22	23 ± 17	160 ± 31*	180 ± 29*
O-1602 (20 mg kg^−1^)	26 ± 16	73 ± 38	191 ± 30*	199 ± 29*

HR [tachycardic response – area under the curve (arbitrary units)]	Control	+CBD	+AM281	+CBD and AM281
Vehicle	96 ± 36	–	–	–
O-l602 (5 mg kg^−1^)	61 ± 24	68 ± 11	39 ± 11	53 ± 22
O-1602 (10 mg kg^−1^)	18 ± 8	79 ± 29	40 ± 12	37 ± 32
O-1602 (20 mg kg^−1^)	72 ± 13	98 ± 32	56 ± 26	38 ± 16

Baseline MABP’s and HR’s for each group were control (146 ± 5 mmHg and 305 ± 10 bpm; *n* = 6); CBD (141 ± 5 mmHg, 296 ± 8 bpm; *n* = 6); AM281 (153 ± 6 mmHg and 296 ± 9 bpm; *n* = 7); and CBD and AM281 (151 ± 5 mmHg and 297 ± 12 bpm; *n* = 7). Values shown are mean ± SEM of the area above/below the curve.

* *P *< 0.05 compared to the same dose in controls. ^*^P < 0.05 versus equivalent 0-1602 dose in control group.

**Figure 6:**
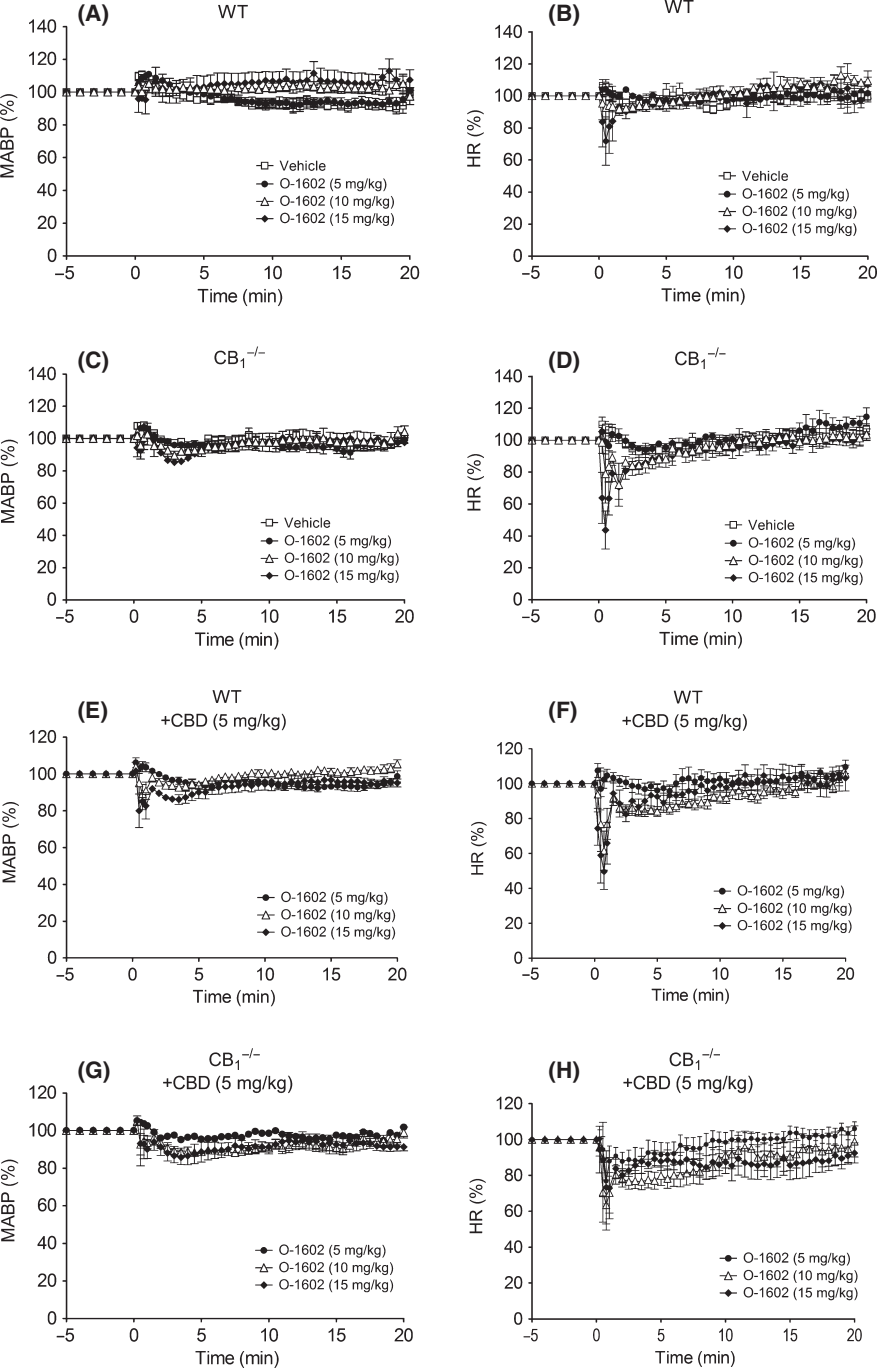
Blood pressure (left hand panels) and heart rate (right hand panels) responses to O-1602 (5–20 mg kg^−1^) in normotensive conscious mice in the absence and presence of CBD (5 mg kg^−1^). Baseline MABP’s and HR’s for each group were WT (128 ± 4 mmHg and 485 ± 9 bpm; *n* = 5; Panels A and B); CB_1_^−/−^ (134 ± 2 mmHg and 438 ± 5 bpm; *n* = 5; Panels C and D); WT with CBD (122 ± 3 mmHg and 384 ± 6 bpm; *n* = 6; Panels E and F); and CB_1_^−/−^ with CBD (135 ± 3 mmHg and 477 ± 5 bpm; *n* = 6; Panels G and H). Values shown are mean ± SEM. Values for areas above/under the curve are shown in Table3.

**Figure 7:**
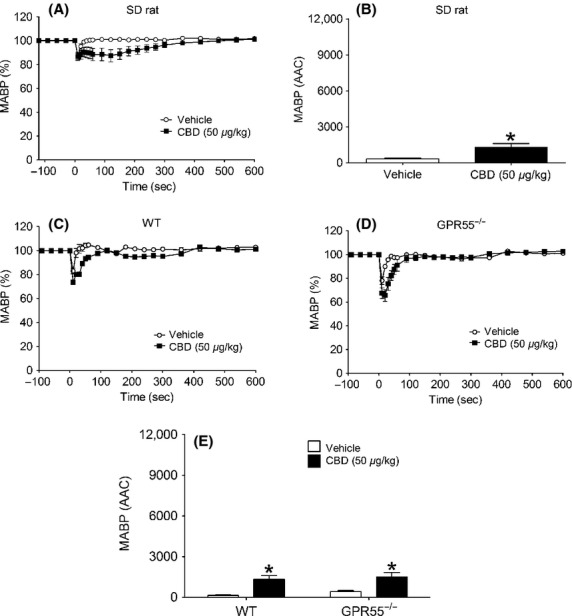
Hemodynamic responses to CBD (50 *μ*g kg^−1^) and its vehicle in normotensive anesthetized rats (A and B) and WT and GPR55^−/−^ mice (C–E). Baseline MABP’s and HR’s for each group were SD Rat (135 ± 4 mmHg and 390 ± 4 bpm; *n* = 8); WT (86 ± 3 mmHg and 323 ± 4 bpm; *n* = 8); and GPR55^−/−^(87 ± 4 mmHg and 329 ± 5 bpm; *n* = 8), respectively. Values shown are mean ± SEM. *P <* *0.05 compared to vehicle response.

In conscious WT mice, O-1602 had no effect on MABP (Fig.5A), but did induce a transient bradycardia at the highest dose tested (Fig.5B; Table3). However, in CB_1_^−/−^ mice a dose-dependent depressor response was observed and the HR responses to O-1602 were markedly enhanced (Figs.6C and D; Table3). Pretreatment with CBD also unmasked a dose-dependent depressor response to O-1602 in WT mice (Fig.6E; Table3) and exacerbated the drug-induced bradycardia (Fig.6F; Table3), but did not further enhance the responses to O-1602 in CB_1_^−/−^ mice (Figs.7G and H; Table2). Finally, administration of O-1602 in the presence of AM281 in GPR55^−/−^ mice did not elicit a depressor response (Table S1).

**Table 3:** Depressor responses to O-1602 in conscious WT and CB_1_^−/−^mice pretreated with metoprolol to remove baroreceptor reflex correction of blood pressure

MABP [depressor response - area above the curve (arbitrary units)]	Control	+CBD	+AM281	+CBD and AM281
Vehicle	99.5 ± 17.8	–	69.5 ± 5.7	–
O-1602 (5 mg kg^−1^)	91.9 ± 38.2	99.6 ± 25.8	64.6 ± 17.5	73.3 ± 24.5
O-1602 (10 mg kg^−1^)	44.1 ± 9.6	73.5 ± 17.8	95.2 ± 6.5	114.6 ± 17.6
O-1602 (15 mg kg^−1^)	61.3 ± 10.9	148.9 ± 17.1†	101.5 ± 4.1*	119.7 ± 18.1

HR [bradycardic response - area above the curve (arbitrary units)]	Control	+CBD	+AM281	+CBD & AM281
Vehicle	44.4 ± 12.2	–	67.7 ± 4.9	–
O-1602 (5 mg kg^−1^)	41.1 ± 20.8	17.4 ± 11.7	55.3 ± 20.9	154.8 ± 61.7
O-1602 (10 mg kg^−1^)	48.7 ± 25.4	186.8 ± 42.1†	130.5 ± 20.5*	298.2 ± 111.7
O-1602 (15 mg kg^−1^)	95.3 ± 6.5*	197.1 ± 17.1†	183.1 ± 50.9*	286.6 ± 105.4

Responses were determined in the absence and presence of CBD (5 mg kg^−1^). Baseline MABP’s and HR’s for each group were: WT (128 ± 4 mmHg and 485 ± 9 bpm; *n* = 5); CB_1_^−/−^ (134 ± 2 mmHg and 438 ± 5 bpm; *n* = 5); WT and CBD (122 ± 3 mmHg and 384 ± 6 bpm; *n* = 6); and CB_1_^−/−^ and CBD (135 ± 3 mmHg and 477 ± 5 bpm; *n* = 6). Values are mean ± SEM.

* *P *<* *0.01 versus vehicle control (within group)

† *P *<* *0.01 versus equivalent dose in WT mice.

### Hemodynamic responses to CBD

**Figure 8:**
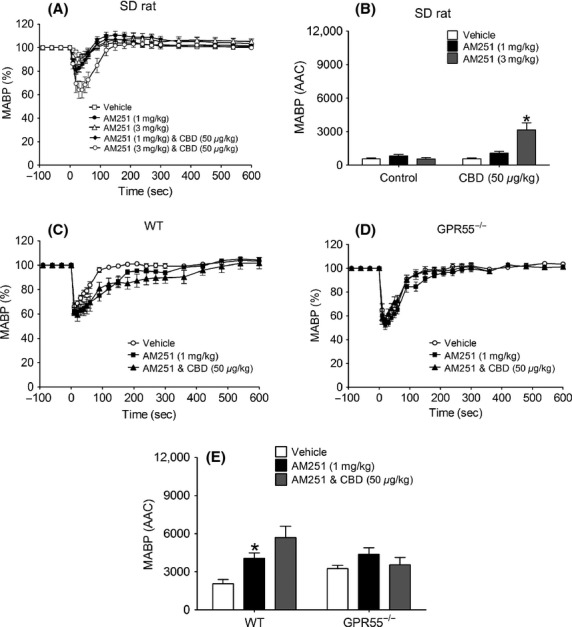
Hemodynamic responses to AM251 (1 and 3 mg kg^−1^) in the absence and presence of CBD (50 *μ*g kg^−1^) in normotensive anesthetized rats (A and B) and WT and GPR55^−/−^ mice (C-E). Baseline MABP’s and HR’s for each group of rats were AM251 alone (123 ± 5 mmHg and 338 ± 6 bpm; *n* = 7); CBD and AM251 (131 ± 2 mmHg and 350 ± 5 bpm; *n* = 8), respectively. Baseline MABP’s and HR’s for each group of WT mice were AM251 alone (87 ± 4 mmHg and 329 ± 6 bpm; *n* = 8); CBD and AM251 (86 ± 3 mmHg and 323 ± 4 bpm; *n* = 8), respectively. Baseline MABP’s and HR’s for each group of GPR55^−/−^ mice were AM251 alone (89 ± 3 mmHg and 339 ± 5 bpm; *n* = 8) and CBD and AM251 (87 ± 3 mmHg and 329 ± 5 bpm; *n* = 8), respectively. Values shown are mean ± SEM. *P <* *0.05 compared to vehicle response.

CBD alone induced a small but measurable and statistically significant depressor response in anesthetized normotensive rats (*P *<* *0.05; Figs.7A and B) and induced depressor responses of a similar magnitude in WT and GPR55^−/−^ mice (Figs.7C–E); HR was unaffected in any experiments (data not shown). AM251 on its own had no effect in anesthetized rats, however, when given in the presence of CBD it induced a depressor response (Fig.8). In contrast, AM251 alone induced a depressor response in WT, but not GPR55^−/−^, mice that was similarly augmented in the presence of CBD (Figs.8C–E).

## Discussion

### GTPγS-binding profiles

The GTP*γ*S-binding assay is a relatively simple and straightforward assay that measures a functional consequence of receptor activation at one of the earliest receptor-mediated events and is used routinely to study receptors coupled to G(i/o) proteins, but can also be used with GPCRs that couple to the G(s) and G(q) families of G proteins (Harrison and Traynor 2003). Since previous studies from our group have shown that activity of ligands at GPR55 and CB_1_ receptors can be measured using this assay (Ryberg et al. 2007), it was an appropriate choice to explore the effects of the ligands under study in CB_1_ and GPR55-transfected cells from both animal species used for the in vivo studies (rat and mouse). Consistent with reports of its action as a selective CB_1_ agonist (Hillard et al. 1999), ACEA demonstrated activity at the CB_1_ receptor in the low nanomolar range. However, ACEA also exhibited agonist activity at GPR55 in the nanomolar range, which to our knowledge is the first demonstration that this ligand also activates GPR55. O-1602 was also found to exert agonist activity at GPR55 in the nanomolar range, with no activity at CB_1_. However, in light of the building evidence to support the notion that O-1602 also activates GPR18 it would have been of interest to also determine the activation profile of O-1602 in cells expressing this receptor.

In terms of the antagonist drugs, CBD showed activity as an antagonist at GPR55, which is consistent with our previous findings (Ryberg et al. 2007), albeit in the high nanomolar range, and did not demonstrate any activity as an agonist at CB_1_ receptors. Finally, as well as exhibiting antagonist activity at CB_1_ receptors in the nanomolar range, AM251 shows clear agonist activity at GPR55 in a similar concentration range, which is consistent with the literature (Pertwee et al. 2010). In contrast, the similar molecule AM281 demonstrated antagonism of CB_1_ at nanomolar concentrations whilst it had no activity as either an agonist or antagonist at GPR55.

### Hemodynamic responses to ACEA

The finding that ACEA caused a marked fall in arterial BP in anesthetized rats, which was blocked by AM251, suggests a straightforward agonist/antagonist relationship and is consistent with the GTP*γ*S-binding data. Most previous studies showing CB_1_-mediated vasodilatation have been undertaken in isolated blood vessel preparations and have used the endocannabinoid, AEA, rather than ACEA, as an agonist. However, ACEA has been shown to decrease coronary perfusion pressure and increase coronary flow (Ford et al. 2002) in isolated perfused hearts, although confirmation of an action at CB_1_ receptors using an appropriate antagonist was not reported. This is therefore the first study to demonstrate a CB_1_-mediated vasodepressor response with ACEA in vivo and that it can be blocked by AM251.

However, while our data support the notion of CB_1_ receptors mediating a vasodepressor effect, it was interesting to note that in anesthetized rats pretreated with CBD, the response to ACEA was also attenuated. If it is assumed that ACEA is a selective CB_1_ agonist, then the most likely explanation for this would be a CB_1_ antagonism by CBD as has been reported in some studies (Pertwee 2008). Furthermore, the inability of AM251 to further prevent ACEA-induced depressor response in the presence of CBD could be through competition with AM251 for the CB_1_ receptor site, since AM251 has a similar binding affinity at CB_1_ (*K*_i_ 7.5 nmol/L; Lan et al. 1999) to that reported for CBD. However, an alternative explanation could be that, since ACEA exerts agonist activity at GPR55 in the nanomolar range, and CBD is an antagonist at this receptor, the effects of ACEA may in fact be mediated by both receptors rather than through CB_1_ alone. In support of this, assuming a total blood volume of ∼20 mL in a 300 g rat, the dose of ACEA administered (3 mg kg^−1^; MW 365.98 g) would be expected to achieve a total (although not necessarily free) blood concentration in the region of 100–120 nmol/L, which is close to the EC_50_ (78 nmol/L) for ACEA activity at GPR55 measured in the GTP*γ*S-binding assay. Therefore, in an attempt to determine whether GPR55 plays a role in the depressor response to ACEA we studied the hemodynamic responses in anesthetized WT and GPR55^−/−^ mice.

In contrast to our findings in rats we were not able to demonstrate a depressor response to ACEA in anesthetized WT mice, the most likely explanation being the significant contribution of the drug vehicle to the response, which was much more marked in mice despite taking significant measures to limit injection artefacts following bolus administration. An alternative explanation is a lack of either vascular CB_1_or GPR55 receptors in mouse resistance vessels, although CB_1_ expression has recently been shown in murine skeletal muscle arterioles (Szekeres et al. 2012) and GPR55 has been shown to be expressed in mouse arteries (Daly et al. 2010). Therefore, whether or not ACEA induces a vasodepressor response, at least in part, through GPR55 remains to be confirmed.

### Hemodynamic responses to O-1602

The second major finding of this study was that, on its own, O-1602 did not induce any changes in BP in normotensive rats or mice, regardless of whether they were anesthetized or in the conscious state. In light of reports that responses to some cannabinoids are only evident (Wheal et al. 2007) or are exaggerated (Ho and Gardiner 2009) in the presence of hypertension, we tested O-1602 in conscious hypertensive rats, but again found no measurable response. However, in conscious hypertensive rats coadministration of the selective CB_1_ antagonist AM281 revealed a marked fall in arterial pressure in response to O-1602, while in conscious WT mice pretreated with CBD, and in CB_1_^−/−^ mice, the highest dose of O-1602 also elicited a depressor response. Taken together these findings suggest that, whatever receptor O-1602 is acting through, it appears to be under CB_1_-mediated inhibition under normal physiological conditions. Although there is increasing evidence that GPR18 is an important receptor target for O-1602 (McHugh et al. 2010), the finding that we could not observe a response to O-1602 in the presence of a CB_1_ receptor antagonist in GPR55^−/−^ mice, combined with the data from the GTP*γ*S activation assay that demonstrated agonist activity of O-1602 at rat and mouse GPR55, suggests that the receptor mediating the depressor response to O-1602 is indeed GPR55. This is in contrast to a study using abnormal cannabidiol (abn-CBD), of which O-1602 is a synthetic analog and has similarly been described as both a GPR55 and GPR18 agonist, in which it was shown to induce a marked vasodepressor response on its own in both WT and GPR55^−/−^ mice, leading to the conclusion that GPR55 does not mediate the depressor responses to abn-CBD (Johns et al. 2007). However, if O-1602 and abn-CBD share a similar profile of activity at GPR55 and GPR18, how can the discrepancy between our findings with O-1602 and with abn-CBD be resolved? One simple explanation may be due to injection artefacts induced by the drug vehicle, since in our mice we have taken account of this in measuring the responses to each cannabinoid ligand. In the case of O-1602 we found that the vehicle produced a fall in arterial BP that was indistinguishable from the response to O-1602. In the study by Johns et al. (2007), however, they did not report any data for vehicle-control responses and therefore it is possible that the responses they reported were exaggerated by a vehicle effect. Alternatively, while various compounds have been shown to activate GPR18 they exhibit biased agonism in terms of the downstream signaling pathways that are activated (Console-Bram et al. 2014) and that, at least in a cell line stably expressing GPR18, O-1602 and abn-CBD exhibit different patterns of GPR18 activation. Therefore, the signaling pathways that elicit the relaxation of vascular smooth muscle in response to GPR55 activation may differ between abn-CBD and O-1602.

### Responses to AM251

AM251 on its own produced a small and transient, but significant, depressor response in both anesthetized rats and WT mice. This is the first report of a vasodepressor response to AM251 in vivo, but a recent report has shown AM251 to increase coronary flow in rat isolated hearts (Andrag and Curtis 2013). It is unlikely that this response to AM251 is due to CB_1_ receptor blockade, since inhibition of any constitutive CB_1_ activity in controlling vascular tone would be expected to increase, rather than decrease, BP and that the response would be prolonged. However, the data from the GTP*γ*S-binding assay in the present studies show that in both rat and mouse membranes expressing GPR55, AM251 has agonist activity with EC_50_ values in the nanomolar range, lending support to other studies showing that AM251 acts as a GPR55 agonist (Ryberg et al. 2007; Kapur et al. 2009). At the doses of AM251 used in the present study (1 and 3 mg kg^−1^; MW 555.24 g), an approximate blood concentration of 60–70 nmol/L would be achieved, which equates approximately to the EC_50_ values from the GTP*γ*S assay. Therefore, it is not unreasonable to suggest that the depressor response to AM251 is mediated via GPR55. Indeed, in support of this we observed a blunted response to AM251 in GPR55^−/−^ mice, although the larger (but not statistically significant) vehicle effect in this strain of mice means that this observation should be interpreted with caution.

In an attempt to further establish a role for GPR55 in mediating the response to AM251, we attempted to block the response to AM251 with CBD. However, instead of an inhibition of the response to AM251 we saw an enhanced response implying that an action of CBD at a different receptor site (possibly CB_1_) is somehow unmasking further the GPR55-stimulating effect of AM251. Indeed, there is increasing evidence of coexpression and “cross-talk” between cannabinoid receptors (reviewed in Pertwee et al. 2010) that supports the notion that ligands binding to the CB_1_ receptor could influence the response to ligands acting through GPR55, and vice versa. For example, expression of both CB_1_ (Hogestatt and Zygmund 2002) and GPR55 (Daly et al. 2010) receptors has been shown in vascular smooth muscle and endothelium, which may lead to possible receptor dimerization. In terms of receptor cross-talk, GPR55 signaling is inhibited in the presence of CB_1_, while CB_1_ receptor signaling is enhanced when GPR55 is coexpressed in the same cells (Kargl et al. 2012). It has similarly been shown in endothelial cells that when CB_1_ and GPR55 receptor integrins are un-clustered, the intracellular signaling pathway activated by AEA are different from those activated when the integrins are clustered, due to CB_1_ receptor uncoupling (Waldeck-Weiermair et al. 2008). In vitro functional evidence to support this concept arises from observations that some cannabinoid ligands (including AM251 and rimonabant) can either inhibit or enhance the downstream signaling following activation of GPR55 by lysophosphatidylinositol (LPI), now regarded as the endogenous ligand for GPR55 (Kotsikorou et al. 2011). Our current study is, we believe, the first functional in vivo evidence of an interaction of this nature, at least in terms of cardiovascular hemodynamics, and if CBD is indeed acting as an inhibitor at CB_1_ then altered signaling could explain the enhanced response to GPR55 activation by AM251.

Although the principal purpose of this study was to profile the various ligands for their action at CB_1_ and GPR55 receptors to induce a depressor response, we did make some observations that may help to understand the role that CB_1_ and GPR55 play in physiological cardiovascular control. For example, the effects of O-1602 on HR in conscious WT mice, while on the basis of the observed pharmacology of this compound cannot be attributed to an action at GPR55, is nevertheless intriguing. When given alone, O-1602 caused a small, but significant, bradycardia at the highest dose tested, however, when mice were pretreated with CBD this bradycardic response was markedly exaggerated and evident at much lower doses; an enhanced response to O-1602 was also seen in CB_1_^−/−^ mice, suggesting that CBD may be acting as a CB_1_ antagonist. However, rather than having no effect in the absence of the CB_1_ receptor, CBD blunted the O-1602 response in CB_1_^−/−^ mice, suggestive that in the absence of CB_1,_ CBD is acting at an additional site. A reduced HR could be either through a direct action on the sinoatrial node, or through interference with cardiac sympathetic control, either through central or peripheral sites. We have found no reports of direct actions of cannabinoid ligands on the pacemaker cells of the heart, but cannot rule this out as a site of action. However, since the bradycardic response was only observed in conscious animals, it is likely to be mediated by alterations in autonomic cardiovascular control as it is well documented that anesthesia itself suppresses cardiac sympathetic outflow. Cannabinoids can suppress sympathetic outflow from the nucleus tractus solitarius (NTS) by a CB_1_ and GABA-mediated mechanism (Seagard et al. 2004; Brozoski et al. 2005) and have been demonstrated to induce cardiovascular depression through a combination of both decreased sympathetic and increased vagal outflow (Niederhoffer et al. 2003). Although it remains to be determined whether an action of O-1602 at GPR18 is responsible for producing the bradycardic response, a recent study has demonstrated an important central role for GPR18 in BP control (Penumarti and Abdel-Rahman 2014). However, in light of the fact that CB_1_ antagonism or receptor knockout markedly enhanced the depressive effect of O-1602 on HR, the modulation of the central cardiovascular control centers by cannabinoids may involve an intricate balance between the activities of different receptors.

We also observed that baseline BPs were higher in conscious (but not anesthetized) GPR55^−/−^ (165 ± 2 mmHg) compared to WT (128 ± 4 mmHg; *P *<* *0.01) mice, implying a role for this receptor in normal physiological cardiovascular control and that a fully functioning sympathetic tone is required to see this effect. We also observed that metoprolol, which was used to prevent excessive baroreceptor reflex-mediated correction of BP responses in conscious animals, induced a profound and long-lasting bradycardia in the conscious GPR55^−/−^ mice, suggesting that in these animals there is an upregulated parasympathetic tone that occurs either as a compensatory mechanism in response to the increased basal arterial pressure, or through a loss of some GPR55-mediated contribution to central cardiovascular control. Although GPR55 is widely distributed in the brain, in particular the caudate nucleus and the putamen (Sawzdargo et al. 1999), thus far there have been no reports of GPR55 expression in the NTS; therefore, it is not possible to speculate to any great extent as to whether or not central GPR55 receptors play a role in cardiovascular control. Second, the raised BP (presumably due to increased peripheral resistance) and profound response to withdrawal of cardiac *β*-adrenoceptor stimulation may be indicative of an altered (heightened) sensitivity of adrenoceptors, suggestive of some interaction between these receptors and GPR55. Indeed, we have recently shown that GPR55^−/−^ mice exhibit a reduced inotropic response to *β*-adrenoceptor stimulation (Walsh et al. 2014). Clearly, therefore, the potential role of GPR55 in cardiovascular control requires further detailed study.

## Abbreviations

abn-CBD: abnormal cannabidiol
ACEA: arachidonyl-2′-chloroethylamide
AEA: anandamide
AM251: 1-(2,4-dichlorophenyl)-5-(4-iodophenyl)-4-methyl-*N*-(piperidin-1-yl)-1*H*-pyrazole-3-carboxamide
AM281: 1-(2,4-dichlorophenyl)-5-(4-iodophenyl)-4-methyl-*N*-4-morpholinyl-1*H*-pyrazole-3-carboxamide
BSA: bovine serum albumin
CB_1_: cannabinoid receptor 1
CB_2_: cannabinoid receptor 2
CBD: cannabidiol
EDTA: ethylenediaminetetraacetic acid
GPR55: G protein coupled receptor 55
HEPES: 4-(2-hydroxyethyl)-1-piperazineethanesulfonic acid
LPI: lysophosphatidylinositol
TRPV1: transient receptor potential vanilloid 1 receptor

## Supporting Information

**Table S1.** Depressor responses to O-1602 in conscious WT and GPR55^−/−^mice pretreated with metoprolol to remove baroreceptor reflex correction of blood pressure. Responses were determined in the absence and presence of AM281 (10 mg kg^−1^). Baseline MABP’s and HR’s for each group were: WT (142 ± 5 mmHg and and 393 ± 6 bpm; *n* = 5); and GPR55^−/−^ (165 ± 2 mmHg* and 436 ± 8 bpm; *n* = 7; **P *<* *0.05 compared to WT mice), respectively. Values are mean ± SEM.
