# Supplementary material for: Pharmacological profiling of the hemodynamic effects of cannabinoid ligands: a combined in vitro and in vivo approach
Source: Pharmacol Res Perspect. 2015 May 8;3(3):e00143. doi: 10.1002/prp2.143 (PMC4492759; doi:10.1002/prp2.143)

Supplementary Material:

**Supplementary Table 1:** Depressor responses to O-1602 in conscious WT and GPR55^-/-^mice pretreated with metoprolol to remove baroreceptor reflex correction of blood pressure. Responses were determined in the absence and presence of AM281 (10mg kg^-1^). Baseline MABP’s and HR’s for each group were: WT (142±5mmHg&393±6bpm; n=5); and GPR55^-/-^ (165±2mmHg* &436±8bpm; n=7; **P*<0.05 compared to WT mice), respectively. Values are mean ± s.e.m.


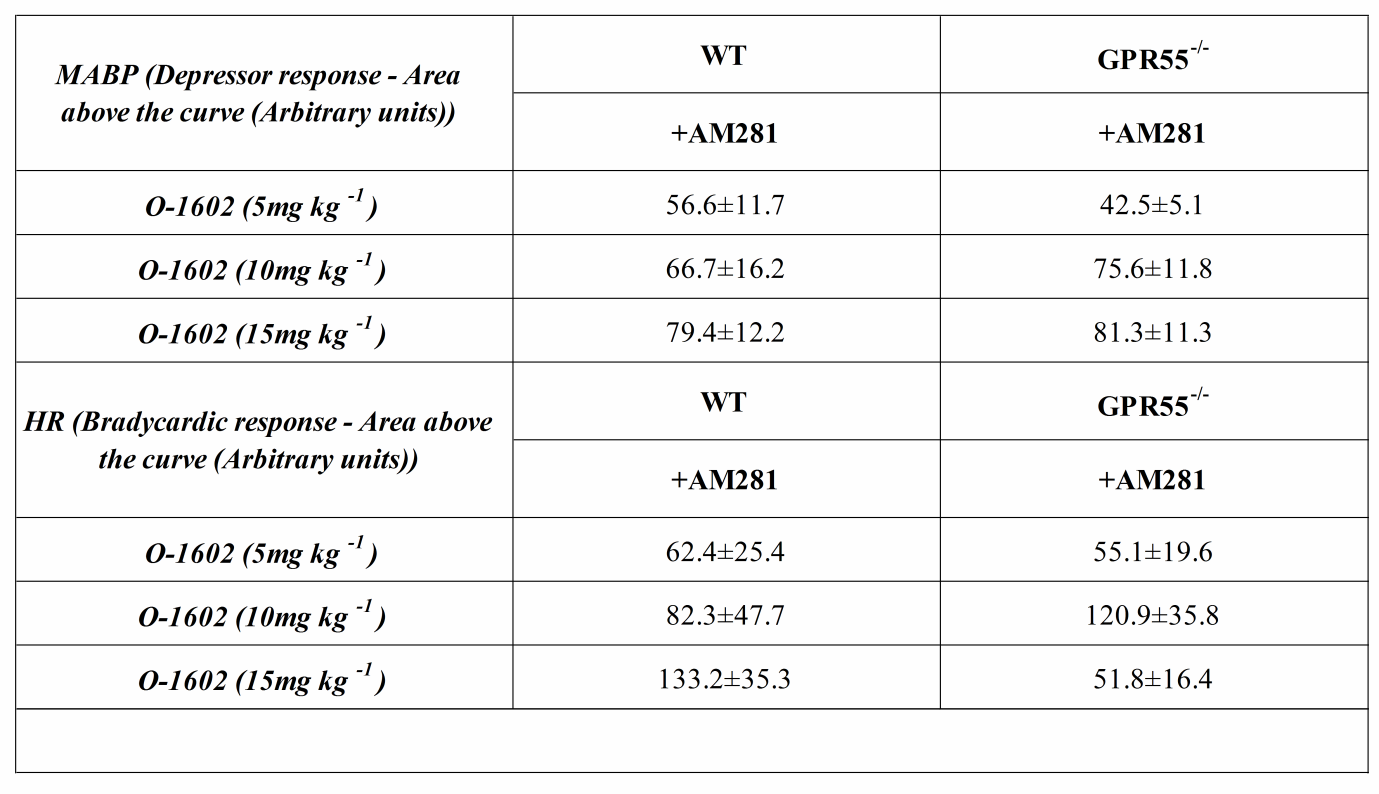

Supplement: Supplementary file 1 [file prp20003-e00143-sd1.docx]
